# Spontaneous Tumor Lysis Syndrome in Prostate Cancer

**DOI:** 10.7759/cureus.18078

**Published:** 2021-09-18

**Authors:** João Pina Cabral, Joana Coelho, Jorge Fortuna, Adriano Rodrigues

**Affiliations:** 1 Internal Medicine, Centro Hospitalar e Universitário de Coimbra, Coimbra, PRT

**Keywords:** tumor lysis syndrome, prostate cancer, solid tumor, low turnover, oncologic emergency, spontaneous tumor lysis syndrome

## Abstract

Tumor lysis syndrome (TLS) is an oncological emergency that most frequently occurs in hematological and high-turnover solid neoplasia. Its incidence in solid, slowly proliferating neoplasia is unclear, primarily because of few published case reports. TLS may be triggered by chemotherapy or infection, or may spontaneously arise. Here, we present a review of the literature and a case of a 58-year-old male patient with prostate cancer who developed spontaneous TLS.

## Introduction

Tumor lysis syndrome (TLS), which is considered an oncological emergency, is characterized by hyperuricemia, hyperkalemia, hyperphosphatemia, and hypocalcemia [1]. TLS occurs more frequently in hematological and fast-growing bulky neoplasia, although it is rare in solid tumors, and spontaneous cases are less common. The prognosis of TLS associated with solid tumors is worse than when present in hematological neoplasia [2]. Here, we report a case of TLS in a 58-year-old man with prostate cancer. We describe how we diagnosed and treated TLS and review the current relevant literature. This case report was presented by the main author as oral communication in the Portuguese National Congress of Internal Medicine.

## Case presentation

We encountered a 58-year-old man who was admitted to our emergency department (ED) with localized right hypochondriac pain. In the ED, the patient underwent abdominal ultrasound testing, which showed multiple lesions suggesting metastatic neoplasia. He was admitted for further testing and pain management. One year earlier, the patient was diagnosed with prostate cancer (PC) with metastatic lesions in his axial skeleton and has since been treated with leuprorelin.

**Figure 1:**
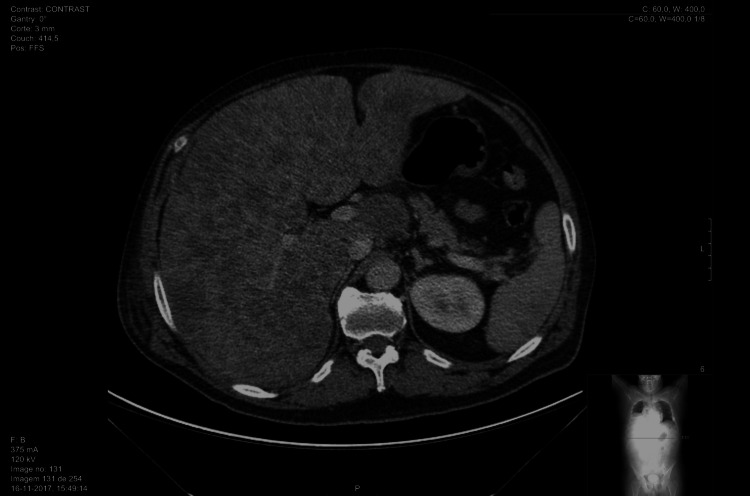
Full-body CT scan showing hepatic lesions.

The patient presented with worsening pain, requiring continuously increasing doses of analgesics while he was waiting to undergo imaging procedures. Full-body computed tomography (CT) showed no evidence of a synchronous primary neoplasm, although multiple secondary hepatic lesions were detected (Figure 1). The patient’s fragile condition prevented invasive biopsy for histological testing.

On the second day of hospitalization, the patient experienced sudden neurological depression, with a Glasgow Coma Scale of 10, which prompted new blood tests (Table 1). The new onset of hyperuricemia, hyperkaliemia, hyperphosphatemia, and acute kidney failure accompanied by increased creatinine and oliguria led us to consider the diagnosis of TLS according to the revised Cairo-Bishop classification (Table 2). Fluid reposition, rasburicase, and sevelamer, along with empirical piperacillin/tazobactam, against Pseudomonas infections, were initiated. The next day, despite analytical improvement (Table 1), the patient succumbed to his illness.

**Table 1:** Blood analyses.

	Blood analysis		
Metabolite or electrolyte	Baseline	1st day	2nd day
Uric acid (mg/dL)	4	15	1
Potassium (mEq/L)	4,46	4.94	4.8
Phosphorus (mg/dL)	3.84	8.39	8.05
Calcium (mmol/L)	2.50	2.29	2.02
Creatinine (µmol/L)	82	184.9	326

**Table 2:** Cairo-Bishop classification for tumor lysis syndrome. LTLS, laboratory tumor lysis syndrome; ULN, upper limit of normal.

Laboratory tumor lysis syndrome - abnormality in two or more	
Metabolite or electrolyte	Diagnostic criteria
Uric acid	≥8 mg/dL or 25% increase from baseline
Potassium	≥6 mEq/L or 25% increase from baseline
Phosphorus	≥6.5 mg/dL (children), ≥4.5 mg/dL (adults), or 25% increase from baseline
Calcium	≥25% decrease from baseline
Clinical tumor lysis syndrome	
LTLS and at least one of the following: (1) creatinine × 1.5 ULN (age >12 years or age-adjusted); (2) cardiac arrhythmia or sudden death; and (3) seizure	

## Discussion

TLS is an oncological emergency characterized by hyperuricemia, hyperkalemia, hyperphosphatemia, and hypocalcemia, and is caused by massive destruction of neoplastic cells [1,3]. Further, acute kidney failure is a common consequence, and metabolic acidosis may occur [4]. High-turnover hematological malignancies such as Burkitt lymphoma and acute lymphoblastic leukemia are common causes of TLS [5], and regular electrolyte and metabolite monitoring is required during treatment. TLS develops during or shortly after the initiation of therapy using glucocorticoids, hormonal agents, or chemotherapeutics such as rituximab [6]. In contrast to hematological neoplasia, solid tumors seldomly present with TLS, and the spontaneous development of the latter is exceptional [7]. Moreover, the prevalence of TLS in solid tumors is difficult to establish because of few published case reports [8]. Although the development of TLS in hematological neoplasia may be anticipated before initiating therapy, this does not apply to solid tumors [9]. For example, TLS is a rare complication of PC [1]. Mirrakhimov et al. presented a case series of TLS in solid tumors, with only five associated with PC [10].

TLS that occurs in patients with solid tumors and spontaneous TLS (STLS) in conjunction with all tumors are associated with increased mortality [7,9]. Most cells proliferate in hematological tumors, and the ensuing large cell mass harbors identical genetic abnormalities, which confer the same theoretical sensitivity to chemotherapy. In contrast, the pathophysiological mechanisms of TLS in solid tumors are not fully understood. Possible mechanisms, which initiate the rapidly ensuing inflammatory cascade, include tissue necrosis from deficient vascularization caused by rapid uncontrolled growth or increased tissue pressure (mostly on the liver and bone) [7].

Patients at risk for developing TLS should be evaluated before treatment according to analyses of the complete blood count, serum electrolytes and metabolites, and urine. Hyperuricemia may be present upon initiation of therapy, and renal deposition of uric acid is facilitated by the acidic environment produced by lactic acidosis and dehydration, leading to renal failure. A ratio of urinary uric acid to urinary creatinine >1 suggests urate nephropathy, whereas a ratio of <1 indicates other causes. Hyperphosphatemia and consequent hypocalcemia may cause renal deposition of calcium and exacerbate renal failure. Interestingly, in STLS, rapidly proliferating tumor cells reutilize released phosphorous such that hyperphosphatemia is less common than in TLS [11]. Further, the destruction of tumor cells releases high concentrations of potassium into the bloodstream. Severe renal failure may lead to diminished potassium excretion and fatal arrhythmias.

When TLS develops, the main goal is to normalize the levels of serum electrolytes and metabolites to prevent or ameliorate the severity of acute kidney failure and to prevent fatal arrhythmias. Clinical and analytical reassessment should be performed every four to six hours [11].

## Conclusions

TLS and STLS delay further chemotherapy and directly or indirectly lead to increased mortality. Although infrequently associated with solid tumors, TLS must be considered when treating any patient with cancer, and close monitoring is warranted. The low incidence of TLS in solid tumors explains our insufficient knowledge of its causes, consequences, and therapeutic strategies. For example, the few published studies describe only single or a small series of cases. Further, the incidence of STLS is lower than that of TLS. The present case report highlights both entities as possible complications of a relatively common and slow-growing cancer as well as the importance of early recognition and treatment.
